# Supplementary material for: Wheat TaTIP4;1 Confers Enhanced Tolerance to Drought, Salt and Osmotic Stress in Arabidopsis and Rice
Source: Int J Mol Sci. 2022 Feb 14;23(4):2085. doi: 10.3390/ijms23042085 (PMC8877497; doi:10.3390/ijms23042085)
Supplement: Supplementary file 1 [file ijms-23-02085-s001.zip › ijms-1551136-supplementary.pdf]

International Journal of *Molecular Sciences*

Supplementary Materials

Wheat TaTIP4;1 Confers Enhanced Tolerance to Drought, Salt and Osmotic Stress in *Arabidopsis* and Rice

Yan Wang, Yaqi Zhang, Yinchao An, Jingyuan Wu, Shibin He, Lirong Sun * and Fushun Hao *

State Key Laboratory of Cotton Biology, School of Life Sciences, College of Agriculture, Henan University, Kaifeng 475004, China; wy875643001wy@163.com (Y.W.); 18437905061@163.com (Y.Z.); ayc19980108@163.com (Y.A.); wujingyuan99@163.com (J.W.); sbhe@henu.edu.cn (S.H.)
* Correspondence: sunlr9208@henu.edu.cn (L.S.); Fushun Hao: haofsh@henu.edu.cn (F.H.); Tel./Fax: +86-371-23881387 (F.H.)

Table S1. Primers used in the experiments.

Names	Forward primers (5′~3′)	Reverse primers (5′~3′)
qTaTIP4;1	AGGATTAAAGGATGGTTCGGTG	TCATATTCTTAAAGCTCCTTCTGCA
TaActin	GACTCCTTCATGCGACCCG	CCACGTTACCCTCAAGCCTTAC
TaTIP4;1-Arabidopsis	CCGGAATTCATGGACACCAAGCATGCGGA	TCCCCCGGGCTAAAAGTCCCAACCAAGTA
TaTIP4;1-rice	CGCGGATCCATGGACACCAAGCATGCGGA	CGGGGTACCCTAAAAGTCCCAACCAAGTA
AtRD29A	AGGATTAAAGGATGGTTCGGTG	TCATATTCTTAAAGCTCCTTCTGCA
AtRD29B	CCAACTTCAGTGTTATCTCCATTG	ACTTATTGACGAGATGCTTAATG
AtRD19	ACGAGCAAGACCCAGAAGTT	AGGAACAATCTCCTCCGATG
AtDREB1B	GGCGTTGGCTTTTCAAGATG	AAGTCGGCATCCCAAACATT
AtDREB2A	AAACCTGTCAGCAACAACAGCAGG	TTAAGCCTGCAAACACATCGTCGC
AtDREB2B	CATCAGAGCCAAGACCAAAACC	TGTAGGACCATTGCCTCAGAAC
AtSOS1	TCGGCAGCATGGTTAATGTG	TTGGCTGAAACGAGACCTTGA
AtSOS2	GGATTTCGGACTCAGTGCAT	CCACGGTGGACAAGAAAACT
AtSOS3	GCTTCTTCACGAATCCGAAC	TTGATGAGCGATGGATTCAA
AtMOCA1	ACTTCACGTCATTCACTACACT	CTGAATGGATCGGTAAATGCAG
AtPKS5	TGACGACGTCTGATCGCAAA	AGATGGAGGGGCAAAATGGG
AtNHX1	GACTCCTTCATGCGACCCG	CCACGTTACCCTCAAGCCTTAC
AtCAT1	AAGTGCTTCATCGGGAAGG	CTCCGAAAGCGCTTCAAC
AtActin2	GGAAAGGATCTGTACGGTAAC	TGTGAACGATTCCTGGAC
OsDREB1A	ATGGGCTGGGACCTGTACTA	GCATCGGAAGCCAGAAAGAG
OsDREB2A	ATGTATGGTCCCACAGCACG	ACAACACAGCTGGCCCATTA
OsPIP1;1	ACTACTAAGCTCATCGCCGC	GGTTCCCAAAGGTCCACACT
OsPIP2;1	CTAAGCTAGGTCGGGCATGG	TGGTGCTTGTACCCGATCAC
OsNAC1	GTCAAGACTGATTGGATCATGC	CCAATCATCCAACCTGAGAGA
OsNAC2	TGTGCCGGATTTACAACAAG	CACCATCGGCTTCCTCTG
OsNAC6	TGCTCGGAGCAGGTGCTGTC	GGCTTGCCCCAGTACATGAGG
OsTIP4;1	ACCCCGGTGCACACTCTG	CATGACCAGGCCCTGCAT
OsP5CS1	AAGGTGGGCACTGCAGTTGT	TCCTTAACCTGCTCGCACAGA
OsRAB16C	TTCCCGGCCAGCACTAAAT	AAACTGCACGTACATCACGACAT
OsCATB	GGTGGGTTGATGCTCTCTCA	ATTCCTCCTGGCCGATCTAC
OsPOX1	CATCCCAGCTCCCAACAA	AGACATGCCAATGGTGTGG
OsLEA3	GGCGCAGTACACCAAGGA	ACCTGCTCACTCGCCTGT
OsSOS1	TCTGCAAAGGAGTGCGTCAT	TCATGCTCCCGTACATGCTC
OsNHX1	CTGATGACCAAAGGGAAGAGC	CATTGAAGATGATCGGAGGG

Names	Forward primers (5′~3′)	Reverse primers (5′~3′)
OsHKT1	GCAGCTGGAAGCAGCAATTC	AAACGAGGAGACTGTGACCG
OsActin	GACCTTGCTGGGCGTGAT	GTCATAGTCCAGGGCGATGT

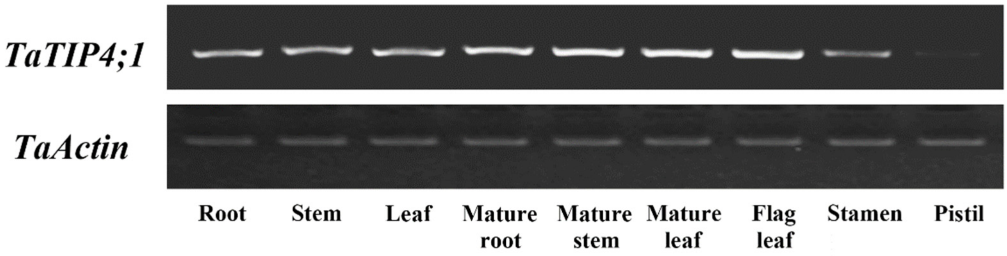

Figure S1. The expression of *TaTIP4;1* in various tissues of wheat. Gene expression was assayed by RT-PCR method, *TaActin* was used as the internal control.

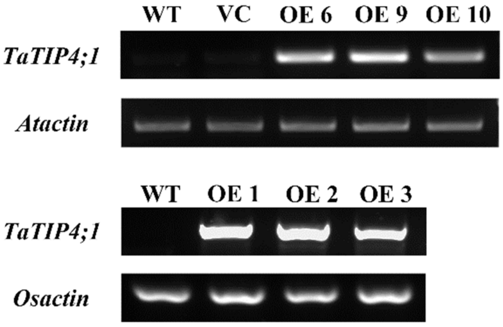

Figure S2. Identification of transgenic plants overexpressing *TaTIP4;1* in *Arabidopsis* and rice. (A) Expression of *TaTIP4;1* in Arabidipsis overexpression line VC (empty vector), and line 6 (OE6), 9 (OE9) and 10 (OE10). *AtActin2* (AT3G18780) was used as an internal control. (B) Transcription of *TaTIP4;1* in rice overexpression line 1 (OE1), 2 (OE2) and 3 (OE3). *OsActin* (AK100267) acted as the internal control.

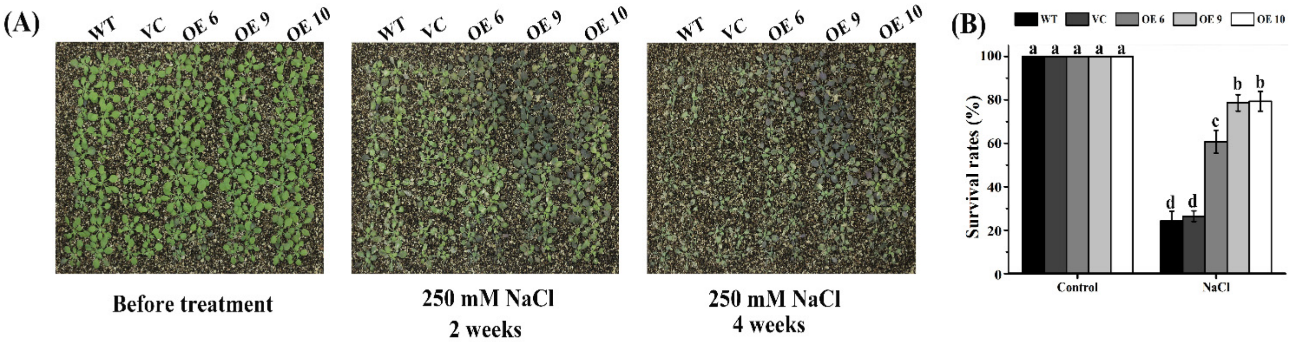

Figure S3. Effects of NaCl on the growth of Arabidopsis WT and *TaTIP4;1* overexpression plants. (A) Seedling growth performances. Ten-old-day seedlings of Arabidopsis WT, VC, OE6, OE9 and OE10 grown in solid 1/2 MS medium were transferred to nutrient soil for 2 weeks. The seedlings were then watered with 250 mM NaCl every 2 d for next 2 weeks or 4 weeks. (B) Survival rates of all plants after treatment with NaCl for 4 weeks in (A). Different lowercase letters above the error bars mean that the values of the plants significantly differed from each other by one way ANOVA and Tukey's HSD test ($P < 0.05$).

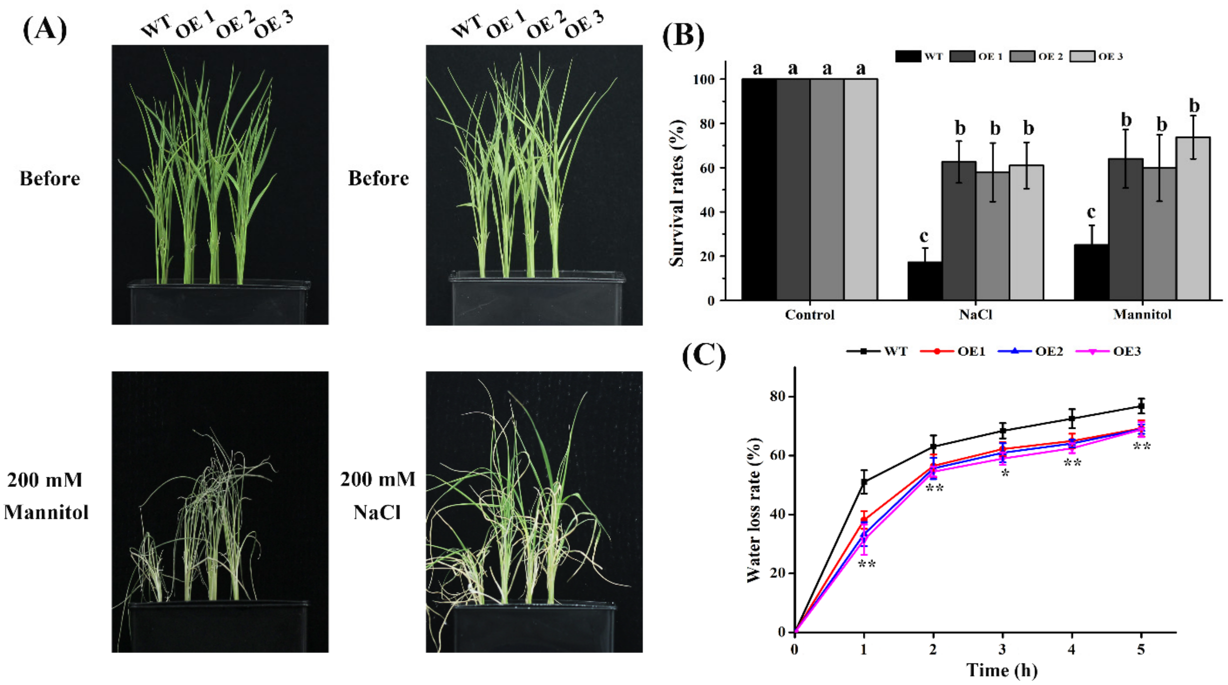

Figure S4. Growth performances of rice WT and transgenic plants in response to NaCl and mannitol stress. (A) Twenty-day-old WT and transgenic plants overexpressing *TaTIP4;1* grown in liquid 1/2 MS medium were treated with or without 200 mM NaCl or 200 mM mannitol for another 10 d. (B) Survival rates of the seedlings upon salt stress in (A). Different lowercase letters above the error bars indicate that the data of the plants markedly differed from each other by one way ANOVA and Tukey's HSD test ($P < 0.05$). (C) Leaf water loss rates of the plants in (A). Single and double asterisk show differences of the data between overexpressors and WT at $P < 0.05$ and $P < 0.01$ levels respectively by student's *t* test.

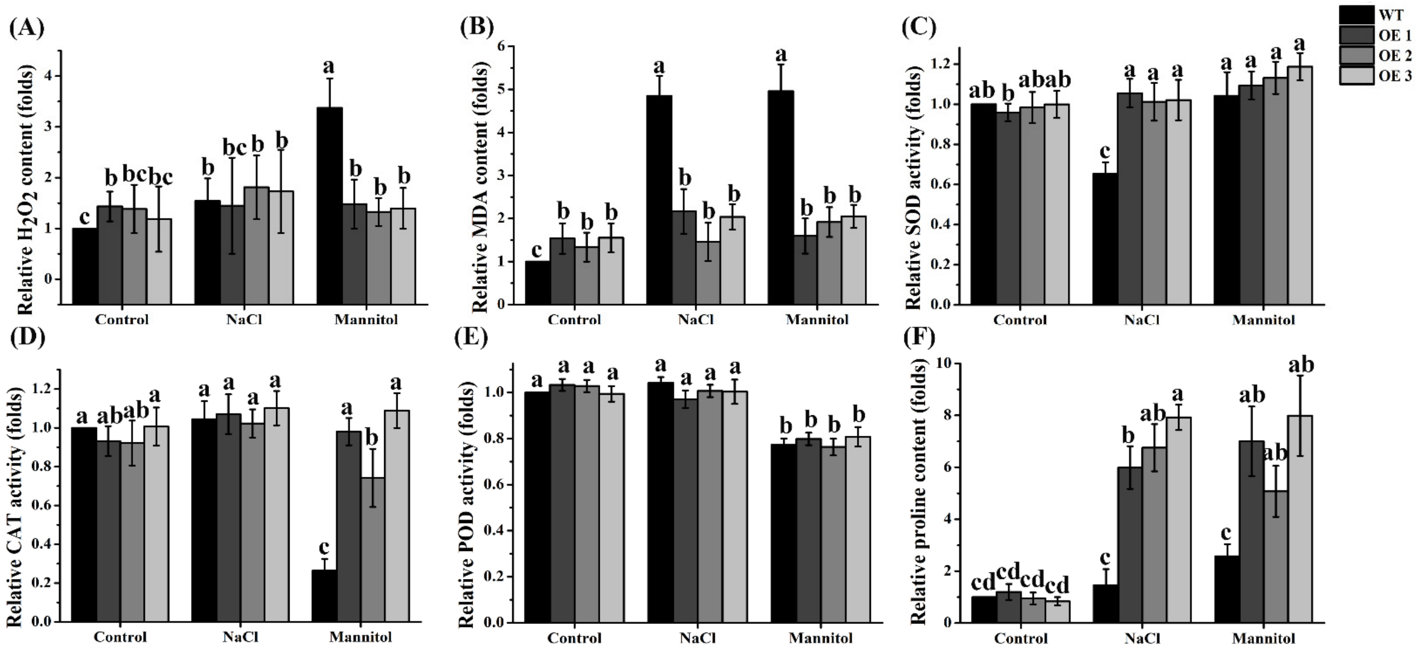

Figure S5. Effects of NaCl and mannitol on the contents of H_2O_2 and MDA, the activity of some antioxidases, and proline accumulation in rice overexpressor of *TaTIP4;1*. Thirty-old day WT and *TaTIP4;1* overexpression seedlings were treated with or without 200 mM NaCl or 250 mM mannitol for 5 d. Levels of H_2O_2 and MDA, the activity of some antioxidases, and proline concentration were

determined. Diverse lowercase letters above the error bars represented that the values of various plants markedly differed from each other by one way ANOVA and Tukey's HSD test ($P < 0.05$).

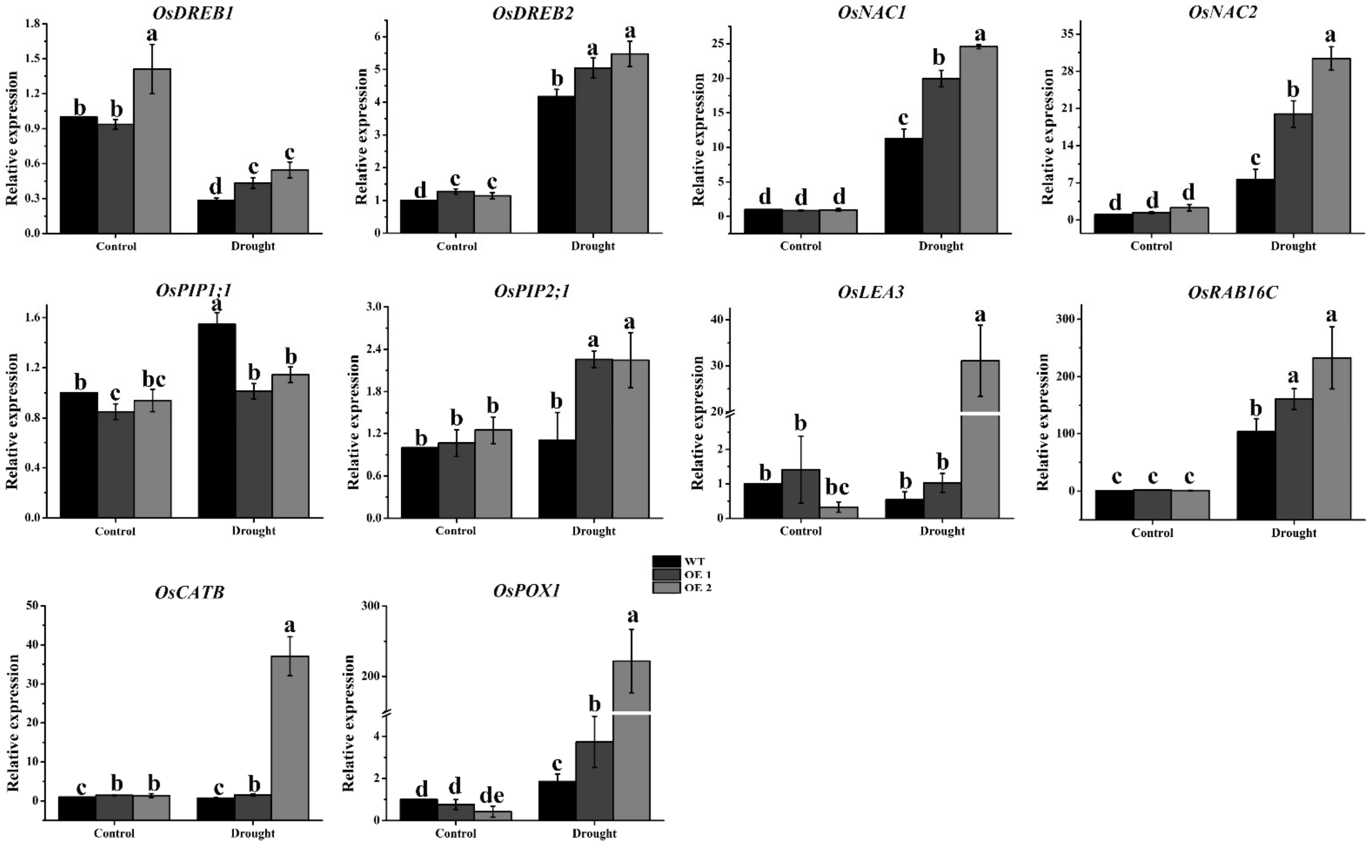

Figure S6. Transcription profiles of some drought responsive genes in rice *TaTIP4;1* overexpressors after water dehydration. Fifteen-old day WT, VC and *TaTIP4;1* transgenic plants were dehydrated for 6 h. Gene expression was detected by qRT-PCR method, *OsActin* was used as the internal control. Diverse lowercase letters above the error bars indicate significant differences in the values among the plants by one way ANOVA and Tukey's HSD test ($P < 0.05$).

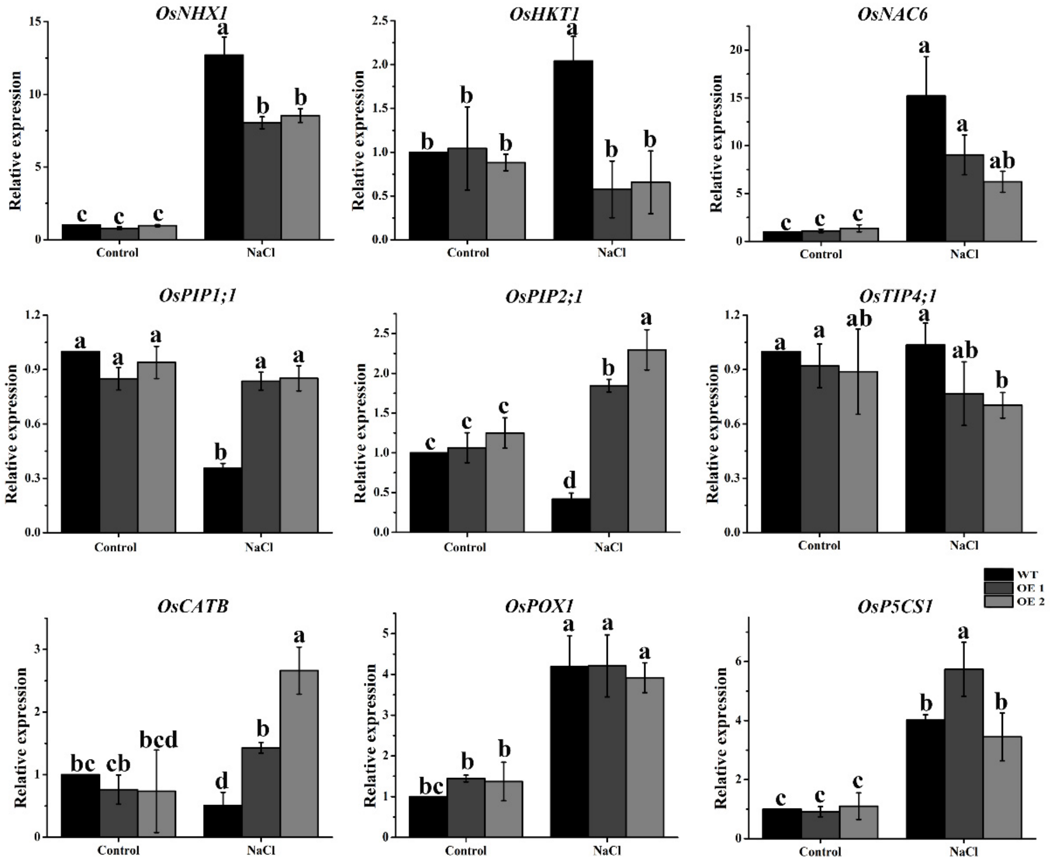

Figure S7. Changes in mRNA abundances of some salt responsive genes in rice *TaTIP4;1* overexpressors after challenged by NaCl. Fifteen-old day WT, OE1 and OE2 plants were treated with or without 200 mM NaCl for 6 h. Gene expression was assayed by qRT-PCR method, *OsActin* was used as the internal control. Diverse lowercase letters above the error bars mean notable differences in the data among various plants by one way ANOVA and Tukey's HSD test ($P < 0.05$).
